# A candidate neuroimaging biomarker for detection of neurotransmission-related functional alterations and prediction of pharmacological analgesic response in chronic pain

**DOI:** 10.1093/braincomms/fcab302

**Published:** 2021-12-22

**Authors:** Daniel Martins, Mattia Veronese, Federico E. Turkheimer, Matthew A. Howard, Steve C. R. Williams, Ottavia Dipasquale

**Affiliations:** Department of Neuroimaging, Institute of Psychiatry, Psychology and Neuroscience, King’s College London, De Crespigny Park, London SE5 8AF, UK; Department of Neuroimaging, Institute of Psychiatry, Psychology and Neuroscience, King’s College London, De Crespigny Park, London SE5 8AF, UK; Department of Neuroimaging, Institute of Psychiatry, Psychology and Neuroscience, King’s College London, De Crespigny Park, London SE5 8AF, UK; Department of Neuroimaging, Institute of Psychiatry, Psychology and Neuroscience, King’s College London, De Crespigny Park, London SE5 8AF, UK; Department of Neuroimaging, Institute of Psychiatry, Psychology and Neuroscience, King’s College London, De Crespigny Park, London SE5 8AF, UK; Department of Neuroimaging, Institute of Psychiatry, Psychology and Neuroscience, King’s College London, De Crespigny Park, London SE5 8AF, UK

**Keywords:** chronic pain, Receptor-Enriched Analysis of Functional Connectivity by Targets (REACT), analgesia, placebo, duloxetine

## Abstract

Chronic pain is a world-wide clinical challenge. Response to analgesic treatment is limited and difficult to predict. Functional MRI has been suggested as a potential solution. However, while most analgesics target specific neurotransmission pathways, functional MRI-based biomarkers are not specific for any neurotransmitter system, limiting our understanding of how they might contribute to predict treatment response. Here, we sought to bridge this gap by applying Receptor-Enriched Analysis of Functional Connectivity by Targets to investigate whether neurotransmission-enriched functional connectivity mapping can provide insights into the brain mechanisms underlying chronic pain and inter-individual differences in analgesic response after a placebo or duloxetine. We performed secondary analyses of two openly available resting-state functional MRI data sets of 56 patients with chronic knee osteoarthritis pain who underwent pre-treatment brain scans in two clinical trials. Study 1 (*n* = 17) was a 2-week single-blinded placebo pill trial. Study 2 (*n* = 39) was a 3-month double-blinded randomized trial comparing placebo to duloxetine, a dual serotonin–noradrenaline reuptake inhibitor. Across two independent studies, we found that patients with chronic pain present alterations in the functional circuit related to the serotonin transporter, when compared with age-matched healthy controls. Placebo responders in Study 1 presented with higher pre-treatment functional connectivity enriched by the dopamine transporter compared to non-responders. Duloxetine responders presented with higher pre-treatment functional connectivity enriched by the serotonin and noradrenaline transporters when compared with non-responders. Neurotransmission-enriched functional connectivity mapping might hold promise as a new mechanistic-informed biomarker for functional brain alterations and prediction of response to pharmacological analgesia in chronic pain.

## Teaser

Martins *et al.* report that patients with chronic knee osteoarthritis pain show altered connectivity in functional circuits related to the molecular systems underlying pain regulation. The functional mapping of these circuits might hold promise as a new neuroimaging biomarker to predict analgesic response.

## Graphical Abstract

**Figure:**
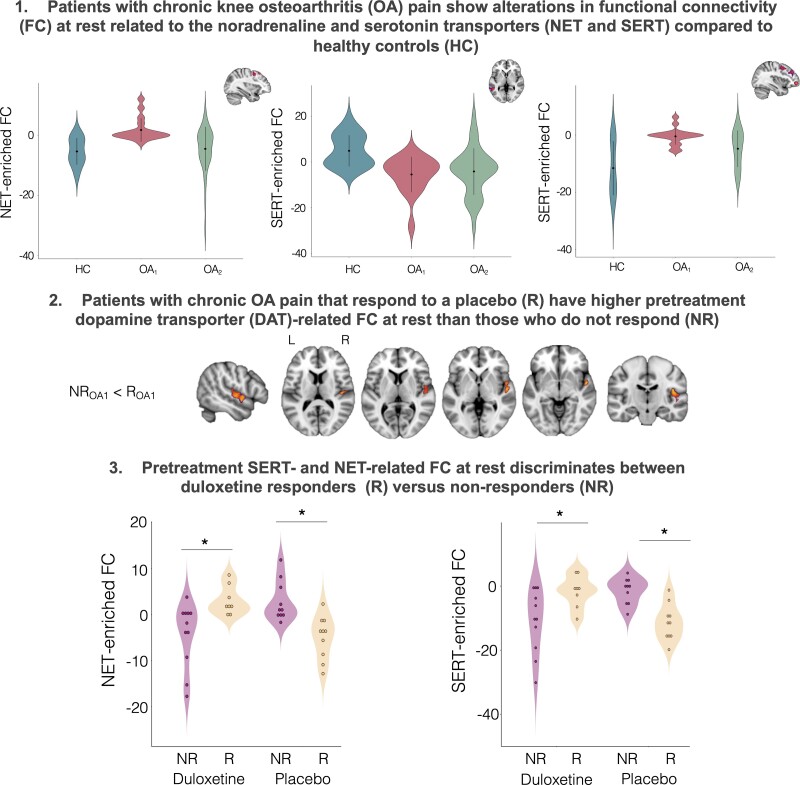

## Introduction

Pain is a world-wide leading cause of disability, constituting one of the primary reasons for people to seek healthcare.^1–3^ Chronic pain is a disease in its own right, characterized by persistence of pain beyond normal healing time.^1^ Despite the high personal and societal costs,^4^ pain management in patients with chronic pain is still generally unsatisfactory.^5^ Although the number of potential pharmacological treatments has grown substantially (i.e. antidepressants, anticonvulsants and opioids),^6^ treatment response is overall low^7^ and why only some patients respond remains poorly understood.^8^ On the contrary, most of the available pharmacological treatments for patients with chronic pain are accompanied by considerable side-effects and risk of misuse (i.e. opioids),^9^ motivating high rates of treatment non-adherence.^10^ A strong case has been made for a mechanism-based and individualized approach to chronic pain therapy^11^; yet, our capacity to predict who may or may not benefit from a specific analgesic treatment is still limited,^12^ leading high numbers of non-responsive patients to experience a range of side-effects with minimal or null clinical benefit. Therefore, developing mechanism-based biomarkers that can guide analgesic treatment selection for chronic pain patients based on the prediction of treatment response remains an unmet target and a clinical need.

Part of this problem stems from our limited understanding of the neurobiological mechanisms underlying chronic pain and, hence, of the mechanisms through which most of these pharmacological treatments might produce persistent pain relief in chronic pain patients.^12^ Currently, it is generally accepted that chronic pain is a multifactorial entity entailing physical, psychological, emotional and social aspects.^1^ Preclinical studies have offered insights into key central mechanisms that might contribute to chronic pain, including sensitization phenomena in an array of nervous system pathways, imbalances in the facilitatory and inhibitory descending modulation pathways from the brain that regulate the transmission of noxious information in the spinal cord, neuroinflammation and glial dysfunction, among others.^13–17^ These findings have fuelled substantial interest in developing neuroimaging-based biomarkers that could unravel how chronic pain affects brain functioning and what form of brain pathophysiology in these patients can be targeted by different treatments.^18,19^ While a range of preliminary diagnosis, prognosis and treatment response brain biomarkers have been suggested (for extensive reviews, see Mackey *et al*.^18^ and van der Miesen *et al*.^19^), to date these biomarkers have provided minimal direct clinical application in the management of chronic pain patients.

Most pharmacological analgesic treatments target specific neurotransmission pathways. For instance, duloxetine, a dual antidepressant often used to manage pain in chronic pain patients, inhibits the reuptake of both serotonin and noradrenaline, increasing their bioavailability in the synapses.^20^ However, neuroimaging-based biomarkers of brain function [i.e. such as those based on measurements of blood oxygenation level dependent (BOLD) signal]^21^ are not specific for any neurotransmission system, limiting the potential mechanistic understanding of how these biomarkers might contribute to explain treatment response. The same limitation applies to the potential neuroimaging biomarkers in unravelling functional changes in neuromodulatory pathways that could guide drug development or repurposing for patients with chronic pain.^12^

This study aims at advancing our understanding on the pain-induced functional alterations in the brain, exploring their link with the neurobiological processes underlying chronic pain and inter-individual differences in analgesic response to pharmacological treatment. Our main hypotheses are that (i) patients with chronic pain present functional alterations in key neurotransmission-related circuits associated with pain control and regulation, when compared with age-matched healthy controls (HCs) and (ii) pre-treatment inter-individual differences in the functional connectivity (FC) of these neurotransmission-related circuits can predict analgesia in response to placebo and pharmacological intervention.

To test these hypotheses, we performed secondary analyses of two openly available resting-state functional MRI (rs-fMRI) data sets^22^ using the recently developed Receptor-Enriched Analysis of Functional Connectivity by Targets (REACT) multimodal framework (Box 1),^23^ which enriches rs-fMRI analysis with information about the distribution density of molecular targets derived from PET and single-photon emission computerized tomography (SPECT) imaging.^23,24^ These data sets included HCs (*n* = 20) and patients with chronic knee osteoarthritis (OA) pain (*n* = 56) who underwent pre-treatment brain scans in two clinical trials. Study 1 (*n* = 17) was a 2-week single-blinded placebo pill trial. Study 2 (*n* = 39) was a 3-month double-blinded, between-subject, randomized trial comparing placebo (*n* = 20) to duloxetine (*n* = 19).

Box 1 Receptor-Enriched Analysis of functional Connectivity by Targets

REACT is a multimodal framework specifically developed to enrich the analysis of fMRI data with information on the spatial distribution of selected molecular systems, using molecular templates derived from PET and SPECT imaging. This integrated analysis allows us to identify functional networks associated with specific molecular systems and explain functional alterations in terms of the underlying molecular substrates of the brain.

**Figure:**
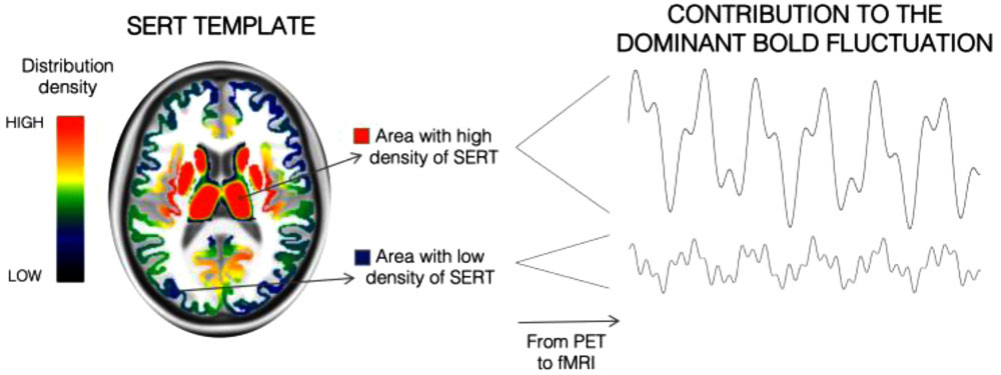


*How it works*: REACT is based on a two-step multivariate regression analysis performed at the subject level. In the first general linear model (GLM), the molecular templates are used as independent variables to inform the model on the spatial configuration of the neurotransmitters under investigation and estimate the dominant BOLD fluctuations of the functional networks related to each molecular system. Regions with high density of neurotransmitters will have a stronger weight in the estimation of the dominant BOLD fluctuation of the corresponding functional network when compared with the regions with lower molecular density. For example, the SERT, which has a high density in the basal ganglia and thalamic regions, will provide a SERT-related functional circuit where those regions will be key centres of the network. On the contrary, regions with a low distribution density of SERT will have a low contribution in the estimation of the functional circuit associated with this neurotransporter.

In the second GLM, the dominant BOLD fluctuations estimated in the previous step are used as independent variables to assess the contribution of each voxel to each dominant BOLD fluctuation associated with the molecular systems under investigation. This analysis will return a set of functional connectivity maps, one for each molecular system being examined.

Of note, while in the first GLM, the areas used as reference regions in the kinetic models for the quantification of the PET/SPECT images are discarded from the analysis because target density in those regions cannot be reliably measured, in the second GLM, every area is taken into account as the BOLD signal is reliably measured in every voxel of the brain. The regions excluded from the first GLM analysis can show either positive or negative functional coupling with the overall receptor-enriched functional network, depending on their positive or negative correlation with the dominant BOLD fluctuation.

*What it means*: When the REACT-based functional maps are compared between groups (e.g. healthy controls versus patients), significant increases or decreases of functional connectivity in brain areas of the functional network related to a specific molecular system can be interpreted as an altered involvement of those areas to the normal functioning of the network. Therefore, this suggests a potential relationship of the functional alterations with the underlying mechanisms involving that molecular system.

*Template choice*: Different types of molecular templates can be used in REACT, according to the specific hypotheses of each study and templates availability. To explore the brain mechanisms underlying a certain disorder, the distribution of neurotransmitter transporters might be more suited to capture the full architecture of a certain system than the distribution of specific receptors, which might not capture the complete picture of neurotransmission related to the specific system. In the case of a drug challenge targeting specific neurotransmitters, the optimal approach would be to use the maps of those specific receptors, if drug binding is known.

We focused our analyses on the functional circuits related to the serotonin transporter (SERT), noradrenaline transporter (NET) and dopamine transporter (DAT), and the µ-opioid receptor, as general indicators of the regional distribution of the neurotransmission related to the serotonin, noradrenaline, dopamine and opioid systems, respectively. When available, the distribution of neurotransmitter transporters is better suited to capture the full architecture of a certain system than the distribution of specific receptors, which might not capture the full picture. We informed our selection of molecular targets by the fact that these neurotransmitters play pivotal roles in pain regulation, namely in those descendent modulatory pathways controlling the spinal transmission of nociceptive information.^25,26^ Furthermore, they have also been implicated in placebo analgesia (i.e. dopamine and opioids)^27^ and correspond to the main molecular targets of duloxetine (i.e. SERT and NET),^20^ whose treatment response was effectively studied herein.

## Methods

### Participants and study design

For this work, we used two openly available rs-fMRI data sets^22^ of patients with chronic knee OA pain who underwent pre-treatment brain scans in two clinical trials. The full details on demographics, inclusion and exclusion criteria have been provided in the original article.^22^ Here, we will simply present a brief summary to help the reader to contextualize. Study 1 was a 2-week single-blinded placebo pill trial where 17 patients with OA [male/female (M/F): 8/9; 56.9 ± 5.7 years] ingested a lactose placebo pill once a day for 2 weeks. Study 2 was a 3-month double-blinded randomized trial in which 39 patients with OA ingested either placebo pills (*n* = 20; M/F: 9/12; 57.6 ± 9.5 years) or duloxetine (*n* = 19; M/F: 9/10; 59.2 ± 4.6 years) at a dose of 30 mg for the first week and escalated to 60 mg for the rest of the treatment period, except for the last week, when the dose was decreased back to 30 mg. In addition, Study 1 also included 20 age-matched HC subjects (M/F: 10/10; 57.9 ± 6.7 years). Of note, the authors of this study were not blinded to the treatment arms.

For Studies 1 and 2, behavioural and clinical parameters were obtained before and after treatment, while brain scans were collected only before treatment. Patients were asked to discontinue their medications 2 weeks before the beginning of the trial and were provided with acetaminophen as rescue medication. All participants gave written informed consent to procedures approved by the Northwestern University Institutional Review Board Committee (STU00039556).

### Behavioural and clinical measures

Patients from both studies completed a general health questionnaire, a Visual Analogue Scale (VAS) on a 0–10 scale for their knee OA pain, the Western Ontario and McMaster Universities Osteoarthritis Index (WOMAC), the Beck Depression Inventory (BDI) and the Pain Catastrophizing Scale (PCS) (note we could only access the raw VAS and WOMAC data). All questionnaires were administered on the day of brain scanning. In Study 2, to partially compensate for regression to the mean effects, VAS was measured three times over a 2-week period prior to the start of treatment and after cessation of medication use, averaged, and used as baseline. Analgesic response was defined *a priori* on an individual basis as at least a 20% decrease in VAS pain from baseline to the end of treatment period; otherwise, subjects were classified as non-responders. This threshold for analgesic response was chosen following the same procedure adopted by the previous study on these data sets.^22^ While the choice of this threshold is arbitrary, we note that a 20% reduction in VAS ratings of pain is higher than the 15% considered to be minimal clinically important.^28^ As a further reference, a 30% reduction in VAS ratings of pain is typically considered a clinical important pain diminution.^29^

### Image acquisition

For all participants in Studies 1 and 2, imaging data were collected with a 3 T Siemens Trio whole-body scanner. A 3D T1-weighted anatomical scan was obtained for each participant using a Magnetization Prepared Rapid Acquisition Gradient Echo acquisition [voxel size = 1 × 1 × 1 mm; repetition time (TR)/echo time (TE) = 2500/3.36 ms; flip angle = 9°; in-plane matrix resolution = 256 × 256; slices = 160; field of view = 256 mm]. Functional MRI data were obtained during rest using a multi-slice T_2_*-weighted echo-planar sequence (TR/TE = 2500/30 ms; flip angle = 90°; number of slices = 40; slice thickness = 3 mm and in-plane resolution = 64 × 64; number of volumes = 300).

### Image pre-processing

The rs-fMRI data sets from Studies 1 and 2 were pre-processed using the FMRIB Software Library (FSL). Pre-processing steps included volume re-alignment with MCFLIRT,^30^ non-brain tissue removal with the Brain Extraction Tool (BET),^31^ an initial spatial smoothing with a 6 mm full width at half maximum (FWHM) Gaussian kernel (size typically recommended for the subsequent de-noising step) and de-noising with an independent component analysis (ICA)-based Automatic Removal Of Motion Artifacts (ICA-AROMA).^32^ Additionally, subject-specific white matter (WM) and CSF masks, obtained from the segmentation of the subjects’ structural images and erosion to minimize the contribution of grey matter partial volume effects, were used to extract and regress out the mean WM and CSF signals from each participant’s pre-processed data set. A high-pass temporal filter with a cut-off frequency of 0.005 Hz was applied, followed by an additional spatial smoothing at FWHM = 6 mm, to obtain a final smoothing of the functional MRI (fMRI) images of ∼8 mm . A study-specific template representing the average T_1_-weighted anatomical image across subjects was built using the Advanced Normalization Tools (ANTs).^33^ Each participant’s data set was co-registered to its corresponding structural scan, then normalized to the study-specific template before warping to standard Montreal Neurologic Institute (MNI)152 space. Images were finally resampled at 2 mm^3^ resolution.

### FC analysis with REACT

For the analysis with REACT, we used the templates of the molecular density distribution of the DAT, NET, SERT and µ-opioid receptor. The DAT map is a publicly available template of ^123^I-Ioflupane SPECT images from 30 HCs without evidence of nigrostriatal degeneration.^34^ The NET atlas is a publicly available template of the [^11^C]MRB PET brain parametric maps from 10 HCs (M/F: 6/4; 33.3 ± 10 years).^35,36^ The SERT atlas is a publicly available template^37^ of [^11^C]DASB PET images of 210 HCs from the Cimbi database.^38^ The µ-opioid receptor map is a publicly available template of [^11^C]Carfentanil PET images of 89 HCs (https://identifiers.org/neurovault.image:115126). All molecular atlases were normalized by scaling the image values between 0 and 1, although preserving the original intensity distribution of the images, and masked using a standard grey matter mask. Of note, for each atlas, we masked out the regions that were used as references for quantification of the molecular data in the kinetic models for the radioligands, namely the occipital areas for DAT, NET and µ-opioid receptor, and the cerebellum for SERT. Finally, we resampled the SERT image to have all atlases in standard MNI space with 2 mm^3^ voxel size.

A detailed explanation of the REACT methodology and its applications can be found elsewhere.^23,24^ In brief, the functional circuits related to the DAT, NET, SERT and µ-opioid receptor systems were estimated using a two-step multivariate regression analysis^39,40^ implemented with the *fsl_glm* command of FSL. In the first step, the rs-fMRI volumes were masked using a binarized mask derived from the molecular atlases to restrict the analysis to the voxels for which the density information of the neurotransmitter was available in the templates. Then, the molecular templates were used as a set of spatial regressors to weigh the rs-fMRI images and estimate the dominant BOLD fluctuation related to each molecular system at the subject level. Those subject-specific time series were then used as temporal regressors in a second multivariate regression analysis to estimate the subject-specific spatial map associated with each molecular atlas. The output consists of four maps per subject, each one reflecting the molecular-enriched FC associated with a specific neurotransmitter. At this stage, the analysis was conducted on the whole grey matter volume. Both data and the design matrix were demeaned (–demean option); the design matrix columns were also normalized to unit standard deviation with the –des_norm option.^39^

### Statistical analysis

We first compared the FC associated with each neurotransmitter system between patients with OA from study 1 (OA_1_) and HC by running exploratory whole-brain two-sample *t*-tests, for each neurotransmission system separately, after controlling for age and gender. For this and all subsequent whole-brain analyses, we applied cluster-based inference within Randomise,^41^ using 5000 permutations per test and contrast, considering a cluster significant if *P*_FWE_ < 0.05, corrected for multiple comparisons using the null distribution of the maximum cluster size across the image. We also applied the Bonferroni correction to correct for multiple comparisons across molecular systems and contrasts investigated. To validate the findings from the exploratory analyses, we then conducted hypothesis-driven analyses comparing patients with OA from Study 2 (OA_2_) and HC. We extracted the mean neurotransmission-enriched FC values in OA_2_ and HC from the clusters where we found significant OA_1_ versus HC differences and compared the two groups. For this and all subsequent hypothesis-driven analyses, we performed frequentist and Bayesian two-sample *t*-tests in SPSS (version 27), controlling for age and gender. Beyond the case–control group differences, we also investigated whether molecular-enriched FC in these clusters would be able to predict the pain ratings (VAS) at baseline in each of the two groups of patients using both frequentist and Bayesian Pearson’s correlations (bootstrapping 1000 samples) in SPSS.

To test whether placebo responders and non-responders differ in pre-treatment molecular-enriched FC, we ran exploratory whole-brain two-sample *t*-tests with Randomise comparing FC related to each system between responders and non-responders from the OA_1_ data set, while accounting for age and gender.

As a further check, we examined whether the molecular-enriched FC differences in OA_1_ could reflect a regression to the mean phenomenon (rather than a placebo pill response) by testing whether FC predicts baseline VAS using frequentist and Bayesian Pearson’s correlations in SPSS.

We then performed a hypothesis-driven analysis in the placebo OA_2_ for those systems showing significant FC differences between placebo responders and non-responders in OA_1_, extracting the mean neurotransmission-enriched FC values in the placebo OA_2_ sub-set from the significant clusters and performing two-sample *t*-tests in SPSS.

Finally, we tested whether different patterns of pre-treatment FC related to neurotransmission underlie differences in response to different analgesic treatments. We investigated this question using the OA_2_ data, which allowed us to examine FC differences between responders and non-responders to placebo and duloxetine. For each functional circuit, we performed a two-way analysis of covariance (ANCOVA) in Randomise to interrogate an interaction effect between treatment type (duloxetine and placebo) and treatment response (responders and non-responders) on FC, controlling for age and gender. For each significant interaction, we extracted the mean FC values within the significant clusters and ran *post hoc* tests in SPSS to evaluate the simple main effects of treatment response within each group separately, controlling for age and gender. Levene’s test was also performed to check the homogeneity of variances. We also calculated frequentist and Bayesian Pearson’s correlations between the mean FC and baseline VAS in the placebo and duloxetine groups separately.

All Bayesian analyses were implemented in JAMOVI, using the default uninformative priors from the software. An increase in Bayes factor (BF) in our analyses corresponds to an increase in evidence in favour of the null hypothesis. To interpret BF, we used the Lee and Wagenmakers’ classification scheme^42^: BF < 1/10, strong evidence for an alternative hypothesis; 1/10 < BF < 1/3, moderate evidence for an alternative hypothesis; 1/3 < BF < 1, anecdotal evidence for an alternative hypothesis; BF > 1, anecdotal evidence for the null hypothesis; 3 < BF < 10, moderate evidence for the null hypothesis; BF > 10, strong evidence for the null hypothesis.

### Data availability

All MRI data of Studies 1 and 2 are available at https://openneuro.org/datasets/ds000208/versions/1.0.0.^43^

### Code availability

The code for performing the REACT-based fMRI analyses is now available as a python package^44^ that can be downloaded from https://github.com/ottaviadipasquale/react-fmri.

## Results

Here, we report in detail only the results of our analyses on molecular-enriched FC and summarize below some of the main findings from the original analyses^22^ on sociodemographic and clinical variables that might help to interpret our novel imaging findings. For a detailed description of sociodemographic and clinical variables, we refer the reader to the original article published elsewhere.^22^

In Study 1, eight patients met the criteria for placebo response and nine patients were classified as non-responders. Responders and non-responders did not differ in baseline pain ratings, age, disease duration, depressive symptoms, pain catastrophizing or medication use at the entry of the study. In Study 2, from those allocated to placebo, 10 patients met the criteria for responders and the other 10 were classified as non-responders. From those allocated to duloxetine, 8 met the criteria for responders and 11 were classified as non-responders. Patients allocated to placebo did not differ in baseline pain ratings when compared with those randomized to duloxetine. In both groups, responders and non-responders did not differ in baseline pain ratings, age, disease duration, depressive symptoms or medication use at the entry of the study. However, in both groups, non-responders showed higher pain catastrophizing than responders. Both placebo and duloxetine produced significant reductions in pain ratings after 3 months of treatment; however, the extent of pain relief did not differ between those treated with placebo and those treated with duloxetine. The clinical variables of knee pain at baseline and pain relief (% analgesia) as measured with the VAS are reported in Supplementary Fig. 1.

### Receptor-Enriched Analysis of Functional Connectivity by Targets

**Figure 1:**
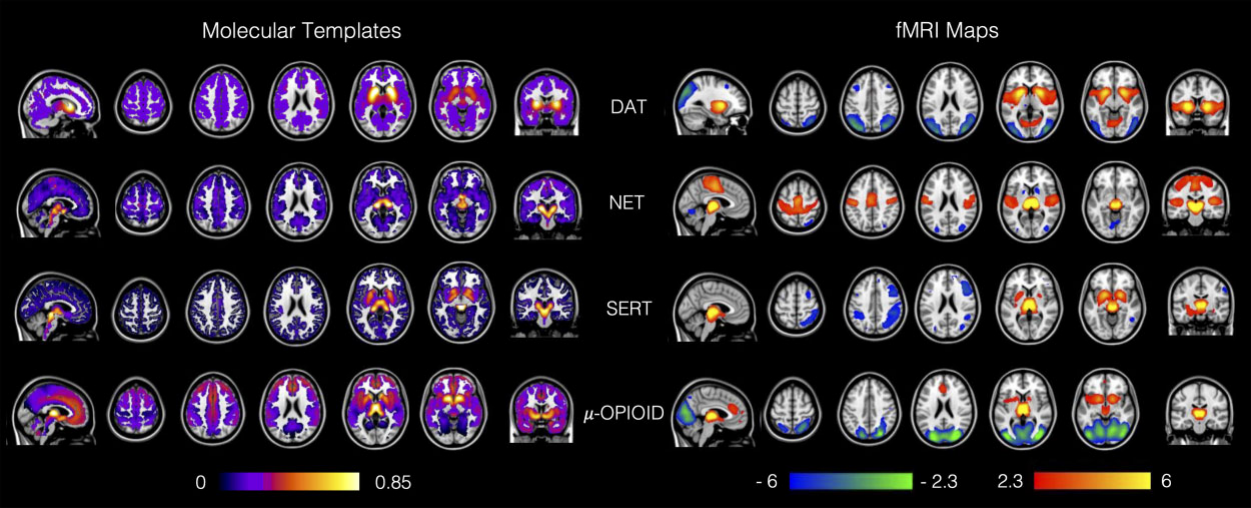
**REACT multimodal framework.** Maps of the molecular templates of the DAT, NET and SERT and the µ-opioid receptor (on the left) and their respective molecular-enriched fMRI maps (on the right). The colour bar on the left represents the molecular density distribution of each template, normalized between 0 and 1 after removing either the cerebellum (for the SERT) or the occipital regions (for the NET, DAT and µ-opioid receptor) as they were used as references for quantification of the molecular data in the kinetic models for the radioligands. The colour bar on the right represents the functional connectivity of each network, expressed in *z*-score. The fMRI maps are averaged across the sub-set of healthy subjects from Study 1.

We used the templates of the molecular density distribution of the DAT, NET, SERT and µ-opioid receptor in the REACT analysis to estimate the corresponding molecular-enriched FC maps of these systems for every subject of the two data sets. In Fig. 1, we provide a summary of the molecular templates (on the left) and their corresponding functional circuits (on the right) estimated by averaging the rs-fMRI maps across HC from Study 1 (for visual purposes only). The resulting maps coherently show high FC values in the core areas of the molecular systems investigated and are in line with the molecular-enriched FC circuits described in previous REACT-based fMRI studies.^23,24,45^

### NET- and SERT-enriched FC differs between patients with OA and HC

**Figure 2:**
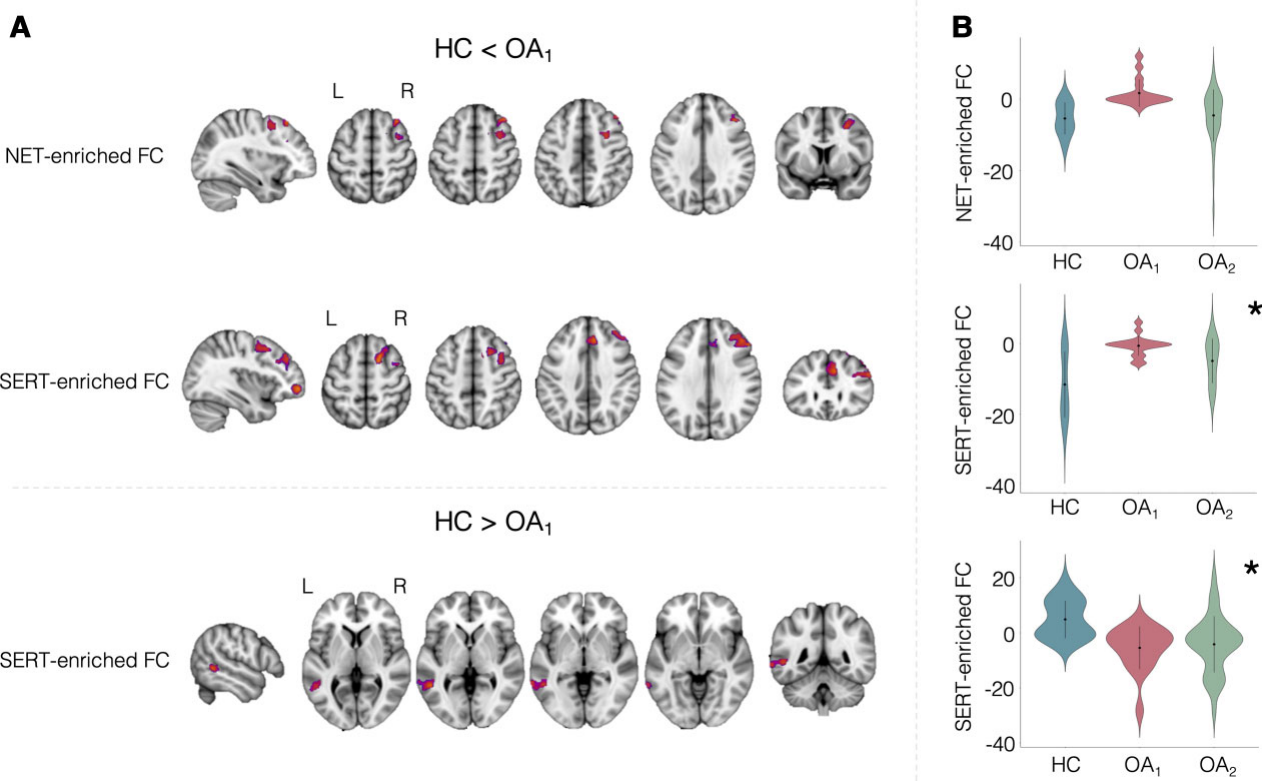
**Alterations in NET- and SERT-enriched FC in patients with chronic knee OA when compared with HC**. (**A**) Whole-brain exploratory analysis on data from Study 1, which identified regions with significantly higher NET- and SERT-enriched FC (top and bottom rows), and other regions with reduced FC in the SERT-enriched functional maps (central row) in OA_1_ patients, when compared with healthy controls. A cluster was deemed significant if it survived *P*_FWE _< 0.05, after correction for multiple comparisons using the null distribution of the maximum cluster size across the image. (**B**) Hypothesis-driven analyses on extracted data from patients in Study 2 (OA_2_) showed a similar pattern of alterations in SERT-enriched FC across the two cohorts. The asterisk denotes significant differences between OA_2_ and HC (*P < 0.05). NET, noradrenaline transporter; SERT, serotonin transporter.

We investigated our first main research question by comparing the FC associated with each neurotransmitter system between OA_1_ and HC. We found significant differences in NET- and SERT-enriched FC (*P*_FWE_ < 0.05) between the two groups (Fig. 2A). Specifically, the OA_1_ group showed increased NET-enriched FC in one cluster including a set of regions spanning the right superior and middle frontal gyrus and the frontal pole [*P*_FWE_ = 0.012; cluster size = 526 voxels; peak *t*-stat value = 4.37; peak MNI coordinates (vox)  =  (29, 76, 64)], and increased SERT-related FC in four clusters including the superior and middle frontal gyri and precentral gyrus [Cluster 1: *P*_FWE_ = 0.002; 889 voxels; peak *t*-stat value = 4.79; peak MNI coordinates (vox)  =  (36, 66, 69)]; the frontal pole and middle frontal gyrus [Cluster 2: *P*_FWE_ = 0.006; 569 voxels; peak *t*-stat value = 4.15; peak MNI coordinates (vox)  =  (19, 78, 52)]; the frontal pole, frontal medial cortex and anterior division of the paracingulate gyrus [Cluster 3: *P*_FWE_ = 0.009; 480 voxels; peak *t*-stat value = 4.47; peak MNI coordinates (vox)  =  (29, 88, 31)]; the paracingulate and superior frontal gyri [Cluster 4: *P*_FWE_ = 0.032; 264 voxels; peak *t*-stat value = 4.26; peak MNI coordinates (vox)  =  (41, 78, 54)]. We also found decreased SERT-enriched FC in the OA_1_ group in one cluster including the superior and middle temporal gyri, supramarginal gyrus and angular gyrus [*P*_FWE_ = 0.032; cluster size = 301 voxels; peak *t*-stat value = 4.14; peak MNI coordinates (vox)  =  (71, 38, 36)]. Of note, only the increase in the SERT-enriched FC survived the Bonferroni correction for multiple comparisons across maps and contrasts. We did not find any group differences in the DAT- and µ-opioid-enriched FC.

To validate these findings, we conducted hypothesis-driven analyses comparing SERT- and NET-enriched FC between OA_2_ and HC. The two-sample *t*-tests showed significant differences in SERT-enriched FC [HC > OA_2_: *F*(3,55) = 7.117, *P* < 0.0005; HC < OA_2_: *F*(3,55) = 5.953, *P* = 0.001], which were similar in direction and magnitude to those observed when we compared OA_1_ and HC. This analysis identified a similar pattern of alterations in SERT-enriched FC across the two cohorts of patients with OA (Fig. 2B). Of note, since the four clusters identified from the contrast HC < OA_1_ in the SERT-enriched FC were localized in overlapping areas, we decided to average the FC values across the clusters and present one global finding and report the results from the single clusters in Supplementary Fig. 2.

The results from the Bayesian analysis of the same data showed that the null hypothesis of a group difference in the NET-enriched FC between HC and OA_2_ was about 3.29 times more likely than the alternative hypothesis (BF_01 _= 3.29, i.e. moderate evidence in favour of the null hypothesis), but 0.0254 and 0.0557 times more likely in the contrasts HC > OA_2_ and HC < OA_2_ in the SERT-enriched FC (i.e. strong evidence supporting the alternative hypothesis), respectively.

Of note, we did not find a consistent pattern of association between NET- and SERT-enriched FC and pain intensity (Supplementary Table 1).

### Placebo responders differ from non-responders in pre-treatment DAT-enriched FC

**Figure 3:**
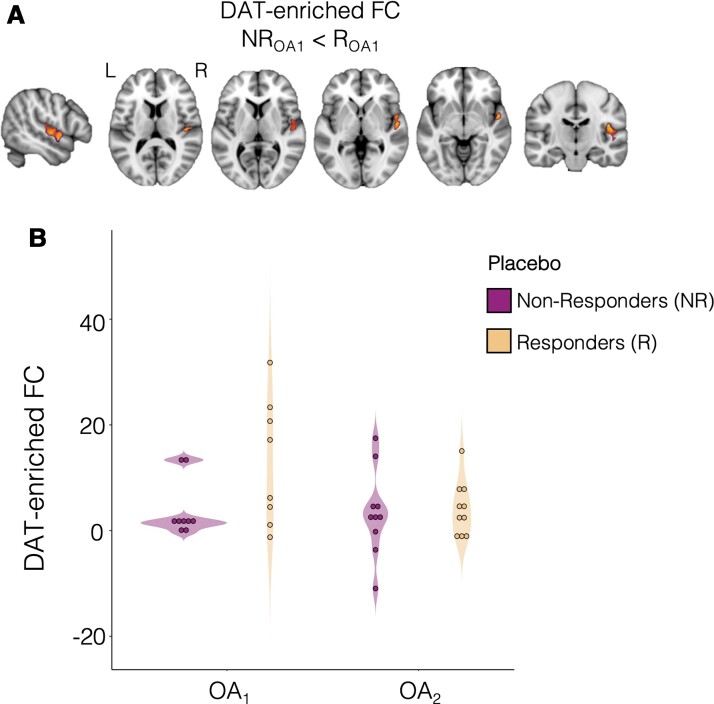
**Differences in pre-treatment DAT-enriched FC between patients with chronic knee OA who responded (R) and did not respond (NR) to placebo administration**. (**A**) Whole-brain two-sample *t*-test conducted on data from Study 1 (OA_1_), which showed significantly higher pre-treatment FC in the DAT-enriched FC in placebo R when compared with NR. A cluster was deemed significant if it survived *P*_FWE _< 0.05, after correction for multiple comparisons using the null distribution of the maximum cluster size across the image. (**B**) Hypothesis-driven analysis on DAT-enriched FC values extracted from the cluster reported in (**A**) in patients from Study 2 did not show any significant differences between placebo R and NR. The violin plots show the mean FC values within the cluster in (**A**) for placebo R and NR in both Studies 1 and 2. OA_1_: *N*_responders _= 8; *N*_non-responders_ = 9; OA_2_: *N*_responders _= 10; *N*_non-responders_ = 10. DAT, dopamine transporter.

We then tested whether placebo responders and non-responders in OA_1_ differ in pre-treatment FC associated with any of the neurotransmitter systems we explored. We found that placebo responders, when compared with non-responders, showed significant increases in DAT-enriched FC in the central and parietal opercular cortex, Heschl’s gyrus, anterior division of the superior temporal gyrus, planum polare and planum temporale [*P*_FWE_ = 0.027; cluster size = 319 voxels; peak *t*-stat value = 3.84; peak MNI coordinates (vox)  =  (20, 58, 36); Fig. 3A]. However, this result does not survive the Bonferroni correction for multiple comparisons across maps and contrasts. No significant differences between placebo responders and non-responders were observed for NET-, SERT- and µ-opioid-enriched FC. Of note, we did not find any correlation between DAT-enriched connectivity and baseline symptom severity (Supplementary Table 2).

Next, we attempted to replicate the DAT-enriched FC findings in a hypothesis-driven analysis using data from OA_2_, but did not find any significant group differences (Fig. 3B). In a Bayesian two-sample *t*-test of the same data, we found that, given the data, the null hypothesis was about 2.46 times more likely than the alternative hypothesis of a group difference (BF_01 _= 2.46, i.e. anecdotal evidence in favour of the null hypothesis).

### Duloxetine responders and non-responders differ in pre-treatment NET- and SERT-enriched FC

**Figure 4:**
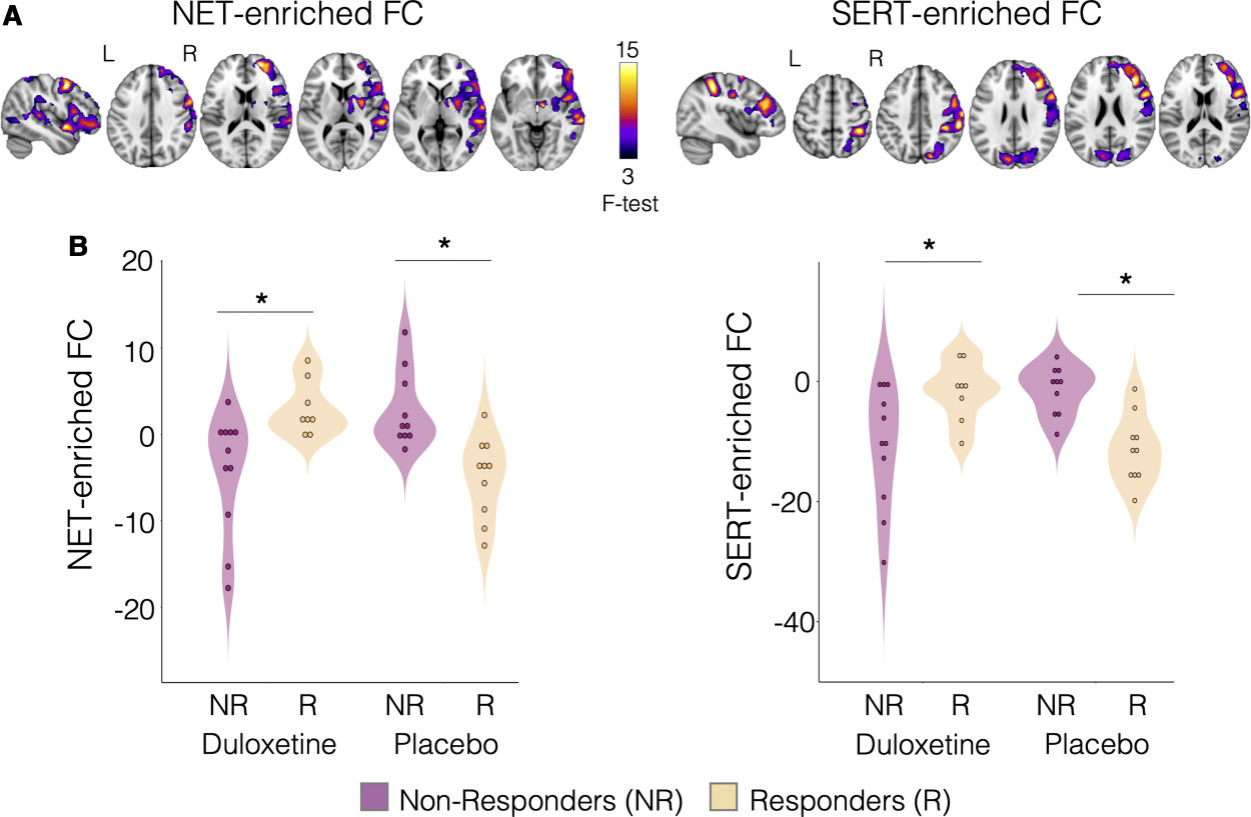
**Treatment type × treatment response interaction in NET- and SERT-enriched FC (Study 2).** (**A**) A two-way ANCOVA showed statistically significant interactions treatment type (duloxetine, placebo) × treatment response (responders, non-responders) in the NET- and SERT-enriched FC maps, after adjusting for patients’ age and gender. A cluster was deemed significant if it survived *P*_FWE _< 0.05, after correction for multiple comparisons using the null distribution of the maximum cluster size across the image. (**B**) *Post hoc* tests were then run on extracted mean FC from those clusters to evaluate the simple treatment response main effects in each treatment group separately, after controlling for age and gender. Significant differences between responders (R) and non-responders (NR) are marked with an asterisk on top of the corresponding violin plots (*P < 0.05 two-tailed, after Tukey’s correction for multiple comparison). Placebo: *N*_responders _= 10, *N*_non-responders_ = 10; duloxetine: *N*_responders _= 8, *N*_non-responders_ = 11. NET, noradrenaline transporter; SERT, serotonin transporter.

Finally, we tested whether different patterns of pre-treatment FC related to neurotransmission underlie differences in response to different analgesic treatments using data from OA_2_. We found significant two-way interaction effects for the NET-enriched FC [*P*_FWE_ = 0.011; cluster size = 14 277 voxels; peak *f*-stat value = 25.5; peak MNI coordinates (vox)  =  (18, 65, 58)] and SERT-enriched FC [*P*_FWE_ = 0.024; cluster size = 14 277 voxels; peak *f*-stat value = 25.5; peak MNI coordinates (vox)  =  (18, 65, 58); Fig. 4A]. In the NET-enriched FC, this interaction spanned the frontal pole, insular cortex, middle and inferior frontal gyrus, precentral gyrus, superior and middle temporal gyrus, postcentral gyrus, supramarginal gyrus and planum temporale. Similarly, in the SERT-enriched FC, the interaction spanned the frontal pole, middle and inferior frontal gyrus, precentral gyrus, postcentral gyrus, superior parietal lobule, supramarginal gyrus, lateral occipital cortex and cuneal cortex. Of note, only the result related to the NET-enriched functional circuit survived the Bonferroni correction for multiple comparisons across maps and contrasts. No significant interaction effects were observed in the DAT- and µ-opioid-enriched FC maps.

We then ran *post hoc* tests to evaluate the simple main effects of response to treatment for the duloxetine and placebo groups separately. In those allocated to receive duloxetine, responders had higher baseline FC in both the NET- and SERT-enriched maps than non-responders; in those allocated to receive placebo, we found the opposite (Fig. 4B). The full statistics resulting from the *post hoc* tests are reported in Supplementary Table 3.

When we investigated a potential regression to the mean phenomenon, we only found a significant negative correlation between SERT-enriched FC and baseline VAS in the placebo group (Supplementary Table 4).

## Discussion

In this study, we investigated whether molecular-enriched FC mapping can provide insights into the brain pathophysiological mechanisms and inter-individual differences in treatment response to pharmacological analgesia during chronic pain. To that end, we applied a novel multimodal approach (REACT) to rs-fMRI data from two studies on patients with chronic pain. We found that, when compared with HC, patients with chronic pain presented FC alterations related to SERT, a key neurotransmission system involved in pain modulation and targeted by drugs currently prescribed to control pain in these patients. These changes were consistent across the two data sets. In line with the known role of dopamine in expectancy and placebo response, we found that pre-treatment DAT-enriched connectivity at rest was higher in patients who responded to a 2-week period of administration of a placebo, when compared with non-responders. We also found that patients who responded to duloxetine, but not those responded to placebo, showed higher pre-treatment NET- and SERT-enriched FC. We discuss each of our main findings below.

Our first main finding was the observation in the discovery data set (Study 1) that chronic pain patients present alterations in NET- and SERT-enriched FC at rest when compared with HC. We partly validated this finding in the replication data set (Study 2), showing a similar pattern of alteration for the SERT-enriched FC, but not for the NET-enriched FC. This lack of replication might be explained by imbalances between cohorts in depression and pain catastrophizing, as reported in the original work of Tétreault *et al*.,^22^ which we could not investigate further due to the unavailability of such data in the open-access repository. Nevertheless, this finding is interesting for two reasons. First, serotonin, together with noradrenaline, is part of the neurotransmission systems involved in pain control and modulation from the brain.^25^ Their role in pain regulation is certainly complex and can encompass both inhibitory (analgesic) and excitatory (hyperalgesic) actions, depending on the site of action, cell type and type of receptor engaged.^25^ Yet, a vast number of studies in animal models have causally implicated alterations in serotonin and noradrenaline neurotransmission in the genesis of persistent pain (for an extensive review, see Mochizucki^46^). For instance, serotonin and noradrenaline depletion through repeated administration of reserpine in rodents is sufficient to induce patterns of persistent tactile allodynia and it has recently been used as a fibromyalgia-like animal model for research on disease mechanisms and drug development.^47–50^ Second, both SERT and NET are targeted by drugs currently prescribed to chronic pain patients, such as tricyclic antidepressants or non-selective inhibitors of the reuptake of serotonin and noradrenaline,^20^ although the understanding of the precise mechanisms through which they might reduce pain is still relatively poor.^20^ The animal literature is strongly supportive of the hypothesis that antidepressants might enhance the engagement of descending inhibitory pain pathways by increasing serotonin and noradrenaline neurotransmission^20^ (though this picture is likely to be more complex given that, for instance, different serotonin receptors can be inhibitory or facilitatory,^51^ or that increases in noradrenaline in brain regions involved in descending pain modulation, such as the dorsal reticular nucleus, can also facilitate pain).^52^ However, whether the same mechanisms are responsible for the clinical effects observed in chronic pain patients has never been explored in depth. Based on our findings, we could speculate that one of the mechanisms through which these drugs might improve pain control in chronic pain patients is through normalizing the alterations in SERT-enriched FC, while an intervention on the NET-enriched FC alterations needs to be further elucidated by studies with larger sample sizes. While we could not test this hypothesis using these data sets since imaging data at follow-up were not collected, we believe this is an interesting question for future work, as it could help to strengthen the rationale for using compounds targeting SERT—and possibly NET—to treat chronic pain.

We did not find any alteration in DAT- or µ-opioid-enriched connectivity in any of the two OA data sets we analysed. This was surprising for several reasons. First, the opioid system has a well-established role in pain regulation^53,54^ and the dopamine system has equally been suggested to be involved in the supraspinal modulation of pain.^55,56^ Second, alterations in opioid and dopaminergic neurotransmission in chronic pain have been reported in human PET studies.^57,58^ For instance, chronic neuropathic pain was shown to be associated with higher striatal dopamine D_2_/D_3_ receptor availability, for which low endogenous dopamine tone is a plausible explanation.^59^ Alterations in μ-opioid receptor availability have been shown across chronic pain conditions,^57^ including arthritis.^60^ Third, opioids figure among the pharmacological agents used to manage chronic pain^61^ and can achieve effective analgesia in at least some types of chronic pain.^62^ Based on these lines of evidence, it would be plausible that chronic pain in OA might be associated with DAT- or µ-opioid-enriched FC changes. While we can only speculate around null findings, we believe at least two factors might have contributed to the lack of DAT- or µ-opioid-enriched connectivity we report here. First, we based our analyses on molecular-enriched FC measured at rest. Hence, we cannot exclude that such alterations might emerge under nociceptive stimulation, which has been shown to recruit the opioid system in human PET studies,^63^ and might enhance case–control differences in µ-opioid-enriched connectivity, if they exist. Second, while one of the exclusion criteria in both data sets was current treatment with monoamine oxidase inhibitors or any centrally acting drug for analgesia and depression, to be eligible patients needed daily pain medication to manage symptoms. While we are unaware of the exact drug class used by these patients before enrolment, it is possible that such treatment might have mitigated potential case–control differences in µ-opioid- or DAT-enriched connectivity, if they existed. This, together with the low sample size of our data sets, might have played a role in the lack of findings on DAT- and µ-opioid-enriched connectivity. Future larger studies will be important to explore these questions further.

Our second main finding was the observation that patients experiencing analgesia in response to a course of 2 weeks of placebo administration (Study 1) present higher pre-treatment DAT-enriched FC (but not SERT-, NET- or µ-opioid-enriched FC) than patients who did not respond. DAT-enriched FC was not related to disease burden prior to the start of placebo treatment in Study 1, diminishing the possibility that the measure is related to regression to the mean rather than a true placebo response. Furthermore, this difference is also unlikely to be confounded by differences between responders and non-responders in disease duration, depressive symptoms, pain catastrophizing or medication use since the groups did not significantly differ in any of these variables (see original study).^22^ This finding suggests that inter-individual differences in DAT-enriched FC might contribute to explaining why patients differ in their responses to placebo. Positive medical responses to placebo treatments are a well-recognized phenomenon observed in many pathologies, particularly for neurological and painful conditions.^64,65^ Analgesia in response to placebo is widely observed in pain clinical trials, in which it often exhibits sustained effectiveness rivalling in magnitude the one from the active treatment.^66,67^ Historically, the placebo effect has been thought of as the end-product of biases in subjective symptom reporting.^68^ This interpretation has evolved through increasing evidence that the placebo effect is mediated by specific neural mechanisms.^67–69^ One of the key theories around the neurobiological mechanisms underlying the placebo effect postulates that it represents a form of reward expectation processing.^70^ Dopamine is thought to be centrally involved in reward expectation and variations from expected outcomes (prediction errors) and has therefore been linked to placebo effects.^71^ For instance, one human PET study has shown that placebo-induced analgesia is associated with decreases in binding [^11^C]raclopride to D2/D3 dopamine receptors in the basal ganglia, possibly reflecting increases in the release of dopamine in these regions.^27^ The same study also reported placebo-induced decreases in [^11^C]carfentanil binding to the µ-opioid receptors, pointing to engagement of the endogenous opioid system during placebo-induced analgesia. However, changes in [^11^C]raclopride binding in the nucleus accumbens emerged as the strongest predictor of placebo-induced analgesia, accounting for 25% of the variance alone. Another study has shown that individual differences in reward response can explain placebo-induced effects and expectations.^72^ The differences in DAT-enriched FC between patients who responded versus those who did not respond to administration of placebo reported here are broadly compatible with this idea. Assuming that higher DAT-enriched FC might be driven by strongest dopamine-related neurotransmission within the dopaminergic circuits (which we index here through DAT density distribution in the brain), then it is plausible that those patients with strongest dopamine-related neurotransmission might benefit the most from expectancy effects, which rely on dopamine release and are at the core of the placebo effect.

This finding was nevertheless not replicated in the hypothesis-driven analysis on data from Study 2, where placebo responders and non-responders did not differ in pre-treatment DAT-enriched FC. The lack of between-group differences was supported by the Bayesian analysis, where the null hypothesis was 2.46 times more likely than the alternative hypothesis. We should highlight though that there is at least one important methodological difference between Studies 1 and 2 that could potentially account for this discrepancy. In Study 1, placebo response was evaluated after 2 weeks of placebo administration, while in Study 2, the placebo protocol lasted for 3 months. Studies have shown that the duration of administration is a determinant of placebo response.^73,74^ Hence, it is possible that while inter-individual differences in DAT-enriched FC are particularly relevant to explain differences in short-term response to placebo, other mechanisms might be involved in the long term. Until further larger studies will revisit these findings, we urge for some caution when interpreting this association between pre-treatment DAT-enriched FC and placebo response. We also note the lack of differences between placebo responders and non-responders on µ-opioid-enriched FC, despite previous evidence that the endogenous opioid system is recruited during placebo-induced analgesia.^27^ However, as explained above, whether that might reflect the fact that FC was measured at rest or that some carry-over effects of previous analgesic treatments biased this result is unclear.

Our third key finding was the observation that patients who responded to duloxetine showed higher pre-treatment NET- and SERT-enriched FC (but not DAT- or µ-opioid-enriched FC) than those who did not respond, while in those patients allocated to placebo, we observed the opposite trend—i.e. lower pre-treatment NET- and SERT-related FC. NET-enriched FC was not related to disease burden prior to the start of placebo or duloxetine treatment, diminishing the possibility that the measure is related to regression to the mean rather than a true response. SERT-enriched FC was also not related to baseline pain ratings in the duloxetine group. Furthermore, these differences are unlikely to be driven by differences between responders and non-responders within each group in disease duration, depressive symptoms or medication use, since the groups did not significantly differ in any of these variables (see original study).^22^ Altogether, these findings suggest that pre-treatment NET- and SERT-related FC at rest might hold promise as a biomarker for duloxetine analgesia response in patients with chronic OA pain.

Duloxetine is a non-selective inhibitor of the reuptake of serotonin and noradrenaline, which enhances their bioavailability at the synaptic level.^20^ The main mechanisms suggested to underlie the analgesic effect observed under duloxetine include enhancement of descending inhibitory pain pathways from the brain through potentiation of serotoninergic and noradrenergic transmission, with consequent inhibition of ascendant transmission of nociceptive inputs from the spinal cord^20^ (although peripheral actions have also received support from some preclinical studies).^75^ This mechanism has received indirect support from a previous study linking response to duloxetine in painful diabetic neuropathy to the integrity of the descending pain inhibitory pathways, as assessed by conditioned pain modulation.^76^ Therefore, the fact that only pre-treatment differences in SERT- and NET-enriched FC exist between duloxetine responders and non-responders matches the pharmacodynamics of the drug and aligns with the basic drug mechanisms through which most likely it induces analgesia. Based on this observation, we suggest that target-enriched FC mapping might open a new avenue in neuroimaging biomarkers of pharmacological treatment response in chronic pain, bringing the advantage of allowing to establish a clearer mechanistic link between the measured neuroimaging biomarker and the neurotransmission-related mechanisms through which a pharmacological treatment targeting a specific neurochemical system might induce analgesia. For instance, since duloxetine acts by inhibiting the reuptake of serotonin and noradrenaline, it is conceivable that its ability to enhance serotoninergic or noradrenergic transmission is moderated by the availability of these neurotransmitters in the synapses, promoting lower accumulation of serotonin/noradrenaline in those where synthesis capacity and tonic release is reduced. Following this line of thought, patients with lower bioavailability of serotonin/noradrenaline, which for this reason might show lower SERT- and NET-related FC connectivity, might not benefit from duloxetine treatment. We should highlight though that at this point any relationship between bioavailability of specific neurotransmitters and target-enriched FC remains speculative and will require validation in further studies.

This empirical observation matches the clinical evidence that non-selective inhibitors of the reuptake of serotonin and noradrenaline might be superior to selective inhibitors of the reuptake of serotonin in promoting analgesia in patients with chronic pain.^20,77^ This superiority is thought to be linked to the fact that antidepressants which also increase the levels of noradrenaline by inhibiting NET might block the spinal transmission of nociceptive input directly through acting on spinal α2 receptors.^20^

Our study has some limitations worth mentioning. First, although REACT improves the specificity of FC analysis, the approach remains relatively indirect and relies on molecular templates estimated in independent cohorts of healthy individuals. Therefore, further specification from intra-regional variation across patients is not possible using the current data set as it would require PET data for each ligand and patient. In any case, it should be noted that although the use of molecular templates of healthy subjects on clinical populations could be seen as a sub-optimal strategy, there would be at least two main disadvantages in using patient-specific templates. First, if the molecular data set used to create the template is not from the same patient cohort, changes in target density for that template might not overlap with the one of the data set under investigation due to factors that might differentially affect the molecular layer (e.g. different stages of pathology, different underlying neurophysiology, comorbidity, etc.). Second, the functional networks estimated from such templates might not properly weight the contribution of the core regions of the molecular systems being examined, eventually resulting in a network that does not reflect the specific features of the molecular system. For these reasons, templates of healthy populations should be adopted in this kind of analyses.

The second limitation of our study is the relatively small sample size of both cohorts. This was in part mitigated by the fact that we attempted to replicate some of our findings in Study 1 using a second cohort of patients from Study 2. However, the issue of small sample sizes was even more prominent in the comparisons between responders and non-responders to placebo and duloxetine. Therefore, while our study provides an important proof-of-concept, future larger studies attempting to replicate our findings will be pivotal. Finally, our findings are restricted to patients with OA and to the prediction of placebo and duloxetine response in this group of chronic pain patients; hence, direct extrapolation to other chronic pain conditions or other pharmacological analgesics should be avoided. Indeed, chronic pain manifests in a range of clinical phenotypes; and even within the boundaries of a specific chronic pain syndrome such as OA, it is likely that different pathophysiological mechanisms are in play in different patients.^78,79^ However, from the findings we gathered in this study, we suggest that future studies expanding this approach to other chronic pain populations and drug classes are worth investing and might be fruitful.

In conclusion, while further clinical validation in larger cohorts is warranted, this study shed light on the functional brain alterations induced by chronic pain and inter-individual differences in brain function that might underlie variability in the response to pharmacological analgesia. The mechanistic insights provided by the molecular-enriched FC mapping might help characterize chronic pain mechanisms, enabling rational and individualized treatment choice. Ultimately, these informed decisions might contribute to decrease unnecessary exposures of patients to ineffective therapies and undesirable side-effects, facilitate treatment adherence and accelerate pain control without long periods of treatment trial-and-error, decreasing the chance that the pain becomes intractable.^80^

## Supplementary Material

fcab302_Supplementary_Data

## Abbreviations

ANCOVA =: analysis of covariance
ANTs =: Advanced Normalization Tools
BDI =: Beck Depression Inventory
BET =: Brain Extraction Tool
BF =: Bayes factor
BOLD =: blood oxygenation level dependent
DAT =: dopamine transporter
FC =: functional connectivity
fMRI =: functional MRI
FSL =: FMRIB Software Library
FWHM =: full width at half maximum
HCs =: healthy controls
ICA =: independent component analysis
ICA-AROMA =: ICA-based Automatic Removal Of Motion Artifacts
MNI =: Montreal Neurologic Institute
NET =: noradrenaline transporter
OA =: osteoarthritis
PCS =: Pain Catastrophizing Scale
REACT =: Receptor-Enriched Analysis of Functional Connectivity by Targets
rs-fMRI =: resting-state functional MRI
SERT =: serotonin transporter
SPECT =: single-photon emission computerized tomography
TE =: echo time
TR =: repetition time
VAS =: Visual Analogue Scale
WM =: white matter
WOMAC =: Western Ontario and McMaster Universities Osteoarthritis Index
