# Supplementary material for: A candidate neuroimaging biomarker for detection of neurotransmission-related functional alterations and prediction of pharmacological analgesic response in chronic pain
Source: Brain Commun. 2021 Dec 22;4(1):fcab302. doi: 10.1093/braincomms/fcab302 (PMC8833258; doi:10.1093/braincomms/fcab302)
Supplement: fcab302_Supplementary_Data [file fcab302_supplementary_data.docx]

**Supplementary Material**

**A candidate neuroimaging biomarker for neurotransmission-related functional alterations and prediction of pharmacological analgesic response in chronic pain**

Daniel Martins, Mattia Veronese, Federico Turkheimer, Matthew A Howard, Steven CR Williams, Ottavia Dipasquale

**Supplementary Figure 1. Clinical measures of knee pain at baseline (top row) and pain relief (% analgesia, bottom row) as measured with the Visual Analogue Scale (VAS) for patients with chronic knee osteoarthritis pain from Study 1 (on the left) and Study 2 (on the right).** Abbreviations: R – Responders; NR – Non-responders.


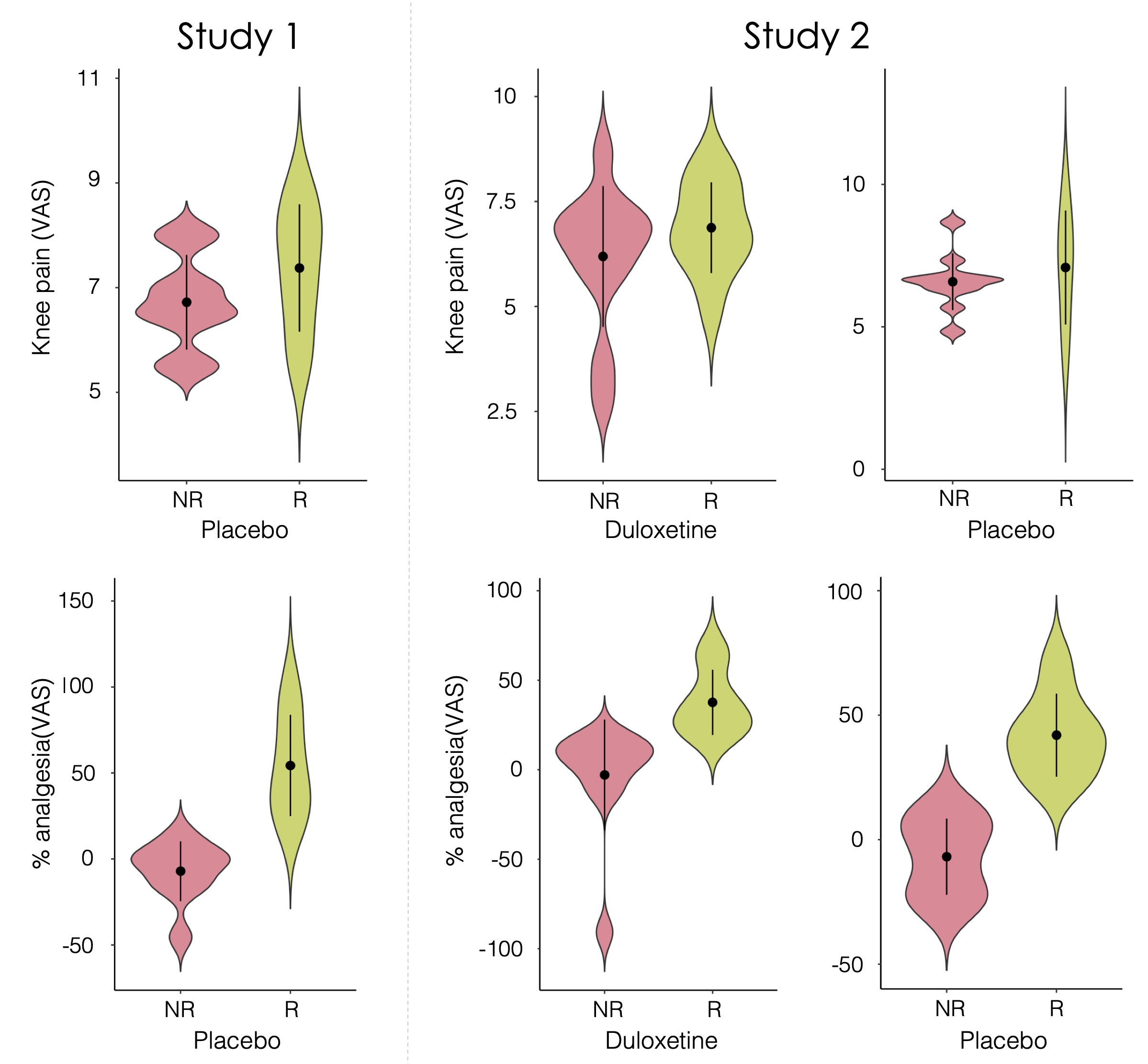


**Supplementary Figure 2. Alterations in SERT-enriched functional connectivity (FC) in patients with chronic knee osteoarthritis (OA) as compared to healthy controls (HC).** Whole-brain exploratory analysis on data from Study 1 identified four clusters with significantly higher SERT-enriched FC in OA1 patients as compared to HC. A cluster was deemed significant if it survived pFWE<0.05, after correction for multiple comparisons by using the null distribution of the maximum cluster size across the image. These clusters were used in a hypothesis-driven analysis on extracted data from patients in Study 2 (OA_2_) and showed a similar pattern of alterations across the two cohorts in clusters 1, 2 and 3 (cluster 1: F(3,55)=11.686, p=0.001; cluster 2: F(3,55)=7.587, p=0.008; cluster F(3,55)=8.345, p=0.006). The asterisk denotes significant differences between OA_2_ and HC. Abbreviations: SERT – Serotonin transporter.


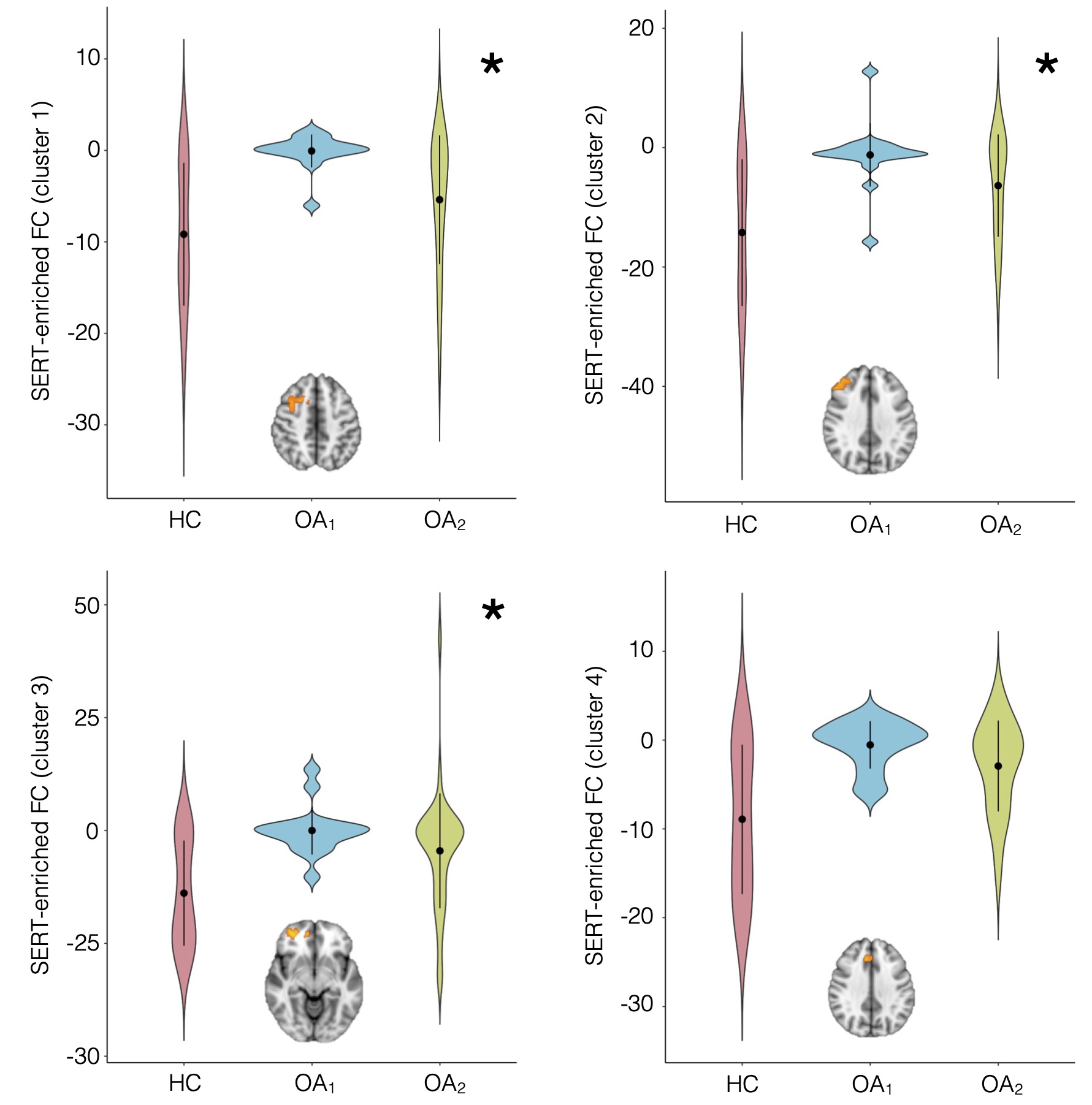


**Supplementary Table 1. Correlations between NET- and SERT-enriched functional connectivity and pain burden.** We calculated frequentist and Bayesian Pearson’s correlations between baseline pain ratings and mean NET and SERT-enriched FC values extracted from the clusters where we found differences between patients and healthy controls in Study 1. We repeated the same procedure for data extracted from patients in Study 2. Statistical significance was set at p < 0.05 (two-tailed). Abbreviations: NET – Noradrenaline transporter; SERT – Serotonin transporter; VAS – Visual analogue scale.

|  | **NET-enriched FC**  **(HC < OA_1_)** | | | **SERT-enriched FC**  **(HC > OA_1_)** | | | **SERT-enriched FC**  **(HC < OA_1_)** | | |
| --- | --- | --- | --- | --- | --- | --- | --- | --- | --- |
| Baseline VAS | **r** | **p-value** | **BF01** | **r** | **p-value** | **BF01** | **r** | **p-value** | **BF01** |
| OA_1_ | 0.227 | 0.381 | 2.34 | 0.170 | 0.514 | 2.74 | -0.001 | 0.998 | 3.34 |
| OA_2_ | 0.225 | 0.096 | 0.881 | 0.323 | 0.015 | 0.468 | 0.227 | 0.092 | 0.708 |

**Supplementary Table 2.** **Correlations between pre-treatment DAT-enriched functional connectivity (FC) and baseline visual analogue scale (VAS) rating of pain in patients from Study 1.** With this analysis, we aimed to examine whether the differences we detected in DAT-enriched FC between placebo responders and non-responders could simply reflect a regression to the mean phenomenon. We calculated frequentist and Bayesian Pearson’s correlations between baseline VAS pain ratings and mean DAT-enriched FC values extracted from the cluster where we found differences between placebo responders and non-responders in the exploratory whole-brain analysis. Statistical significance was set at p < 0.05 (two-tailed). Abbreviations: DAT – Dopamine transporter; FC – Functional Connectivity

|  | **DAT-enriched FC** | | |
| --- | --- | --- | --- |
| **Study 1** | **r** | **p-value** | **BF01** |
| Baseline VAS | -0.025 | 0.923 | 3.32 |

**Supplementary Table 3.** **Simple main effects of treatment response in the duloxetine and placebo groups**. We used a general linear model, considering treatment response (responders, non-responders) as a fixed factor and age and gender as covariates. Statistical significance was set at p < 0.05 (two-tailed), after correction for multiple comparisons with Tukey. Abbreviations: NET – Noradrenaline transporter; SERT – Serotonin transporter; FC – Functional Connectivity.

|  | **NET-enriched FC** | | | **SERT-enriched FC** | | |
| --- | --- | --- | --- | --- | --- | --- |
| **Treatment group** | **F** | **p-value** | **Partial 𝜂^2^** | **F** | **p-value** | **Partial 𝜂^2^** |
| Duloxetine | F(1,15) = 3.76 | 0.034 | 0.43 | F(1,15) = 4.60 | 0.018 | 0.479 |
| Placebo | F(1,16) = 4.45 | 0.019 | 0.455 | F(1,16) = 8.57 | 0.001 | 0.616 |

**Supplementary Table 4.** **Correlations between pre-treatment SERT and NET-enriched functional connectivity (FC) and baseline visual analogue scale (VAS) rating of pain in patients from Study 2.** With this analysis, we aimed to examine whether the differential differences we detected in NET and SERT-enriched FC between placebo and duloxetine responders and non-responders could simply reflect a regression to the mean phenomenon. We calculated frequentist and Bayesian Pearson’s correlations between baseline VAS pain ratings and mean NET and SERT-enriched FC values extracted from the clusters where we found significant interactions between treatment type and treatment response in the exploratory whole-brain analyses, for each treatment type group separately. Statistical significance was set at p < 0.05 (two-tailed). Abbreviations: NET – Noradrenaline transporter; SERT – Serotonin transporter; FC – Functional Connectivity; VAS – Visual analogue scale; BF – Bayes Factor.

|  | **NET-enriched FC** | | | **SERT-enriched FC** | | |
| --- | --- | --- | --- | --- | --- | --- |
| **Study 2** | **r** | **p-value** | **BF01** | **r** | **p-value** | **BF01** |
| Baseline VAS  (Placebo group) | -0.425 | 0.062 | 0.707 | -0.582 | 0.007 | 0.123 |
| Baseline VAS  (Duloxetine group) | -0.287 | 0.233 | 1.81 | 0.027 | 0.914 | 3.50 |
